# Possible mechanism underlying the association between height and vascular remodeling in elderly Japanese men

**DOI:** 10.18632/oncotarget.23660

**Published:** 2017-12-23

**Authors:** Yuji Shimizu, Shimpei Sato, Jun Koyamatsu, Hirotomo Yamanashi, Mako Nagayoshi, Koichiro Kadota, Shin-Ya Kawashiri, Takahiro Maeda

**Affiliations:** ^1^ Department of Community Medicine, Nagasaki University Graduate School of Biomedical Science, Nagasaki, Japan; ^2^ Department of Cardiovascular Disease Prevention, Osaka Center for Cancer and Cardiovascular Disease Prevention, Osaka, Japan; ^3^ Department of Island and Community Medicine, Nagasaki University Graduate School of Biomedical Science, Nagasaki, Japan

**Keywords:** platelets, height, vascular remodeling, CIMT, CD34-positive cell, Gerotarget

## Abstract

Height is reported to be inversely associated with cardiovascular disease. And platelets play an important role in vascular remodeling by supporting CD34-positive cells. To clarify the association between height and platelet, we conducted a cross-sectional study of 219 elderly Japanese men. Since hemoglobin concentration is influenced by vascular remodeling activity, an analysis stratified by hemoglobin level was performed. An inverse association was seen between height and platelet count in subjects with a high hemoglobin concentration (≥ 14.5 g/dL), but not in subjects with a low hemoglobin concentration (< 14.5 g/dL). The standardized parameter estimates (β) were β = −0.22, *p* = 0.019 for subjects with high hemoglobin, and β = −0.01, *p* = 0.931 for subjects with low hemoglobin. We also found a positive association between platelets and carotid intima media thickness (CIMT) and circulating CD34-positive cells in subjects with high hemoglobin (partial correlation coefficient (r) = 0.21, *p* = 0.037 and *r* = 0.40, *p* =< 0.001), but not in subjects with low hemoglobin (*r* = 0.04, *p* = 0.710 and *r* = 0.06, *p* = 0.544). In subjects with a high hemoglobin concentration, platelets were inversely associated with height, and positively associated with CIMT and circulating CD34-positive cells. These results indicate that subjects with a short stature activate vascular remodeling to a much greater extent than subjects with a tall stature.

## INTRODUCTION

Height is reported to be inversely associated with incidence of cardiovascular disease [1]. Our previous study of Japanese men revealed an inverse association between height and carotid atherosclerosis among overweight subjects, but not in those who were not overweight [2]. Atherosclerosis is known to be a chronic inflammatory disease [3], and a higher white blood cell count has also been identified as a marker of systemic inflammatory activity that is known to be associated with atherosclerosis [4]. In connection with these phenomena, we also reported that short stature is associated with inflammatory disadvantages since high white blood cell count is inversely associated with height in subjects with a body mass index (BMI) ≥ 23 kg/m^2^ but not in those with a BMI < 23 kg/m^2^ [5]. Additionally, an Asian-specific inflammation-related single nucleotide polymorphism (SNP) (rs3782886) was also revealed to be significantly associated with short stature [6]. These studies indicate that short stature is associated with vascular pathological disadvantages.

On the other hand, a growing body of evidence indicates that platelets play an important role in inflammation [7], and are also reported to be important in the development of atherosclerotic lesions as an initial actor [8]. Platelets promoted the mobilization of bone marrow-derived CD34-positive cells into the peripheral blood [9]. Furthermore, platelets not only induce differentiation of CD34-positive cells into endothelial progenitor cells and endothelial cells, but also into foam cells [10, 11] which is a well-known contributing factor in the development of atherosclerotic lesions.

Since the development of atherosclerosis is one aspect of vascular repair, and we previously reported that platelet count indicates the activity of vascular repair (endothelial repair and development of atherosclerosis) [12], studies clarifying the association between height and platelet count and its association with circulating CD34-positive cells might be an efficient tool to evaluate the risk of vascular pathological disadvantages caused by short stature.

In addition to the above, bone metabolism is known to be associated with vascular maintenance [13], and age-related reduction in bone marrow activity is known to cause anemia in the elderly [14]. Additionally, hypertension is a strong endothelial impairment factor, and hemoglobin is known to be positively associated with hypertension [15], atherosclerosis [16], and hypertension-induced vascular damage [17]. Therefore, subjects with low hemoglobin concentration should demonstrate lower vascular maintenance activity, while subjects with a high hemoglobin concentration should show higher activity. Since the aim of our present study was to evaluate the influence of height on activated vascular repair (platelet levels) in elderly men, we conducted a cross-sectional study stratified by hemoglobin level of elderly Japanese men aged 65–69 years who participated in a general health check-up from 2013–2015.

## RESULTS

### Characteristics of study population

A simple correlation analysis found no significant correlation between height and age; *r* = −0.11 (*p* = 0.093). Among 219 subjects, 115 presented with low hemoglobin concentration (Hb < 14.5 g/dL) and 104 with high hemoglobin concentration (Hb ≥ 14.5 g/dL).

Characteristics of the study population based on hemoglobin status are shown in Table 1. Compared to those with low hemoglobin, high hemoglobin subjects demonstrated significantly higher values for reticulocytes, white blood cells, systolic and diastolic blood pressure, and body mass index.

**Table 1:** Characteristics of the study population based on hemoglobin levels

	Low hemoglobin Hb < 14.5 g/dL	High hemoglobin Hb ≥ 14.5 g/dL	*p* value
No. of participants	115	104	
Age, years	67.2 ± 1.2	67.5 ± 1.3	0.103
Reticulocytes, ‰	10.4 ± 3.5	12.3 ± 3.5	< 0.001
Circulating CD34 positive cells, cells/μL	1.17 ± 1.92	1.36 ± 1.36	0.419
White blood cells, cells/μL	5161 ± 1316	5869 ± 1368	< 0.001
Platelets, ×10^4^/μL	22.2 ± 6.3	21.4 ± 4.9	0.302
Mean carotid intima media thickness (CIMT), mm	0.69 ± 0.11	0.71 ± 0.12	0.154
Systolic blood pressure, mmHg	130 ± 18	137 ± 16	0.004
Diastolic blood pressure, mmHg	76 ± 12	82 ± 10	< 0.001
Body mass index (BMI), kg/m^2^	21.9 ± 1.8	22.5 ± 1.8	0.021
Serum HDL-cholesterol (HDL), mg/dL	58 ± 14	57 ± 13	0.695
Serum triglycerides, mg/dL	113 ± 110	111 ± 49	0.869
Hemoglobin A1c (HbA1c), %	5.6 ± 0.5	5.8 ± 0.7	0.149
Serum aspartate aminotransferase (AST), IU/L	24 ± 7	26 ± 10	0.144
Serum γ-glutamyltranspeptidase (γ-GTP), IU/L	41 ± 34	48 ± 47	0.179
Serum creatinine, mg/dL	0.85 ± 0.16	0.82 ± 0.15	0.306
Height, cm	164.2 ± 5.0	163.8 ± 6.2	0.563

Age: mean ± standard deviation.

### Correlation analysis of hematological parameters, carotid intima media thickness (CIMT), and height

Table 2 shows a correlation analysis of hematological parameters, CIMT, and height. For subjects with high hemoglobin, platelets were significantly positively associated with CIMT, circulating CD34-positive cells and white blood cells, and inversely associated with height. We also found a significant positive association between reticulocytes and white blood cells and height, but not CIMT

**Table 2:** Correlation analysis of hematological parameters, carotid intima media thickness (CIMT) and height

	Platelets	Reticulocytes	Mean CIMT	Circulating CD34 positive cells	White blood cells	Height
**Low hemoglobin (Hb < 14.5 g/dL)**						
**Simple correlation analysis**						
Platelets	−	0.16 (0.090)	−0.01 (0.928)	0.11 (0.248)	0.45 (< 0.001)	−0.03 (0.723)
Reticulocytes	0.16 (0.090)	−	0.002 (0.985)	0.002 (0.985)	0.15 (0.107)	0.03 (0.722)
Mean CIMT	−0.01 (0.928)	0.002 (0.985)	−	0.08 (0.373)	−0.02 (0.852)	−0.03 (0.753)
**Partial correlation analysis**						
Platelets	−	0.18 (0.070)	0.04 (0.710)	0.06 (0.544)	0.44 (< 0.001)	−0.06 (0.515)
Reticulocytes	0.18 (0.070)	−	0.03 (0.735)	0.002 (0.981)	0.16 (0.100)	0.02 (0.836)
Mean CIMT	0.04 (0.710)	0.03 (0.735)	−	0.07 (0.497)	0.06 (0.516)	−0.01 (0.925)
**High hemoglobin (Hb ≥ 14.5g/dL)**						
**Simple correlation analysis**						
Platelets	−	0.13 (0.179)	0.23 (0.017)	0.35 (< 0.001)	0.36 (< 0.001)	−0.25 (0.010)
Reticulocytes	0.13 (0.179)	−	0.09 (0.387)	0.14 (0.151)	0.42 (< 0.001)	0.28 (0.004)
Mean CIMT	0.23 (0.017)	0.09 (0.387)	−	−0.02 (0.815)	0.28 (0.004)	0.06 (0.552)
**Partial correlation analysis**						
Platelets	−	0.08 (0.466)	0.21 (0.037)	0.40 (< 0.001)	0.31 (0.002)	−0.24 (0.018)
Reticulocytes	0.08 (0.466)	−	0.16 (0.120)	0.10 (0.324)	0.38 (< 0.001)	0.37 (< 0.001)
Mean CIMT	0.21 (0.037)	0.16 (0.120)	−	−0.01 (0.925)	0.26 (0.010)	0.10 (0.357)

CIMT: carotid intima media thickness. Partial correlation analysis is adjusted for age, SBP, BMI, triglycerides, HDL, AST, γ-GTP, HbA1C, and creatinine. Circulating CD34-positive cells, triglycerides, γ-GTP, and creatinine are calculated in logarithm values.

### Association between platelets and height

From a simple correlation analysis, height was found to be significantly inversely associated with platelets in subjects with high hemoglobin but not in subjects with low hemoglobin (Table 3).

**Table 3:** Simple correlation coefficient of platelets and other variables

	Low hemoglobin Hb < 14.5 g/dL		High hemoglobin Hb ≥ 14.5 g/dL	
	r	*p*	r	*p*
No. of participants	115		104	
Age	−0.16	0.098	0.01	0.934
Systolic blood pressure	0.06	0.517	0.02	0.859
Body mass index (BMI)	0.06	0.536	−0.08	0.424
Serum HDL-cholesterol (HDL)	−0.08	0.378	−0.08	0.395
Serum triglycerides	0.02	0.873	0.09	0.376
Hemoglobin A1c (HbA1c)	−0.03	0.740	0.13	0.174
Serum aspartate aminotransferase (AST)	−0.26	0.006	−0.15	0.142
Serum γ-glutamyltranspeptidase (γ-GTP)	−0.16	0.079	0.16	0.101
Serum creatinine	−0.014	0.884	−0.21	0.029
White blood cells (WBC)	0.25	0.008	0.30	0.002
Height	−0.03	0.723	−0.25	0.010

r: simple correlation coefficient. Triglycerides, γ-GTP, and creatinine are calculated in logarithm values.

**Figure 1:**
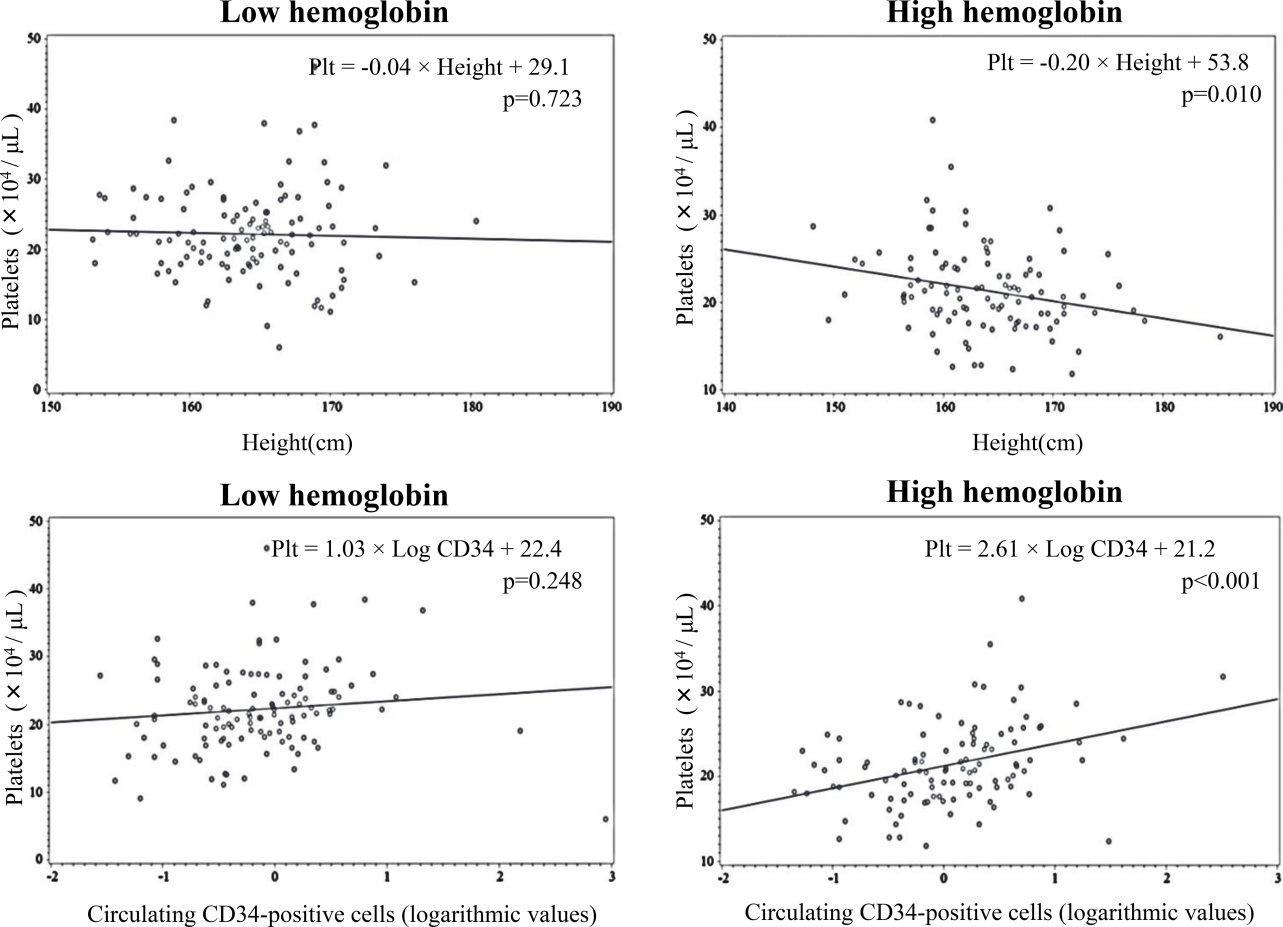
Scatter plot of platelets and height, and platelets and circulating CD34-positive cells

Scatter plot data indicated a significant inverse association between platelets and height and a significant positive association between platelets and circulating CD34-positive cells in subjects with high hemoglobin but not in subjects with low hemoglobin (Figure 1).

These associations remained unchanged even after further adjustment for known cardiovascular risk factors (Table 4).

**Table 4:** Multiple linear regression analysis of platelet with relevant factors adjusted for confounding factors

	Low hemoglobin Hb < 14.5 g/dL			High hemoglobin Hb ≥ 14.5 g/dL		
	Β	β	*p*	Β	β	*p*
No. of participants		115			104	
Age	−0.19	−0.04	0.695	0.26	0.07	0.445
Systolic blood pressure	0.05	0.14	0.114	−0.01	−0.02	0.853
Body mass index (BMI)	0.12	0.03	0.707	−0.22	−0.08	0.414
Serum HDL-cholesterol (HDL)	0.03	0.07	0.510	0.00003	0.0001	1.000
Serum triglycerides	1.23	0.11	0.287	0.05	0.004	0.971
Hemoglobin A1c (HbA1c)	−1.56	−0.13	0.153	0.37	0.05	0.612
Serum aspartate aminotransferase (AST)	−0.16	−0.18	0.064	−0.14	−0.30	0.005
Serum γ-glutamyltranspeptidase (γ-GTP)	−1.53	−0.16	0.112	1.69	0.22	0.054
Serum creatinine	−2.27	−0.06	0.490	−3.64	−0.13	0.176
White blood cells (WBC)	0.002	0.44	< 0.001	0.001	0.30	0.002
Height	−0.01	−0.01	0.931	−0.17	−0.22	0.019

Β: parameter estimate. β: standardized parameter estimate. Triglycerides, γ-GTP, and creatinine are calculated in logarithm values.

## DISCUSSION

The major findings of the present study are a significant inverse association between platelet count and height, and a significant positive association between platelet count and circulating CD34-positive cells and CIMT in subjects with high hemoglobin concentration. No such significant associations were present in subjects with low hemoglobin concentration.

In a previous study, we reported an inverse association between height and high white blood cell count in those with a BMI ≥ 23kg/m^2^ but not with a BMI < 23 kg/m^2^ [5]. Since a high white blood cell count, which indicates elevated inflammatory activity, is significantly positively associated with atherosclerosis [4], height may be inversely associated with this disease. In fact, our previous study of Japanese men revealed an inverse association between height and carotid atherosclerosis in overweight (BMI ≥ 25 kg/m^2^), but not non-overweight (BMI < 25 kg/m^2^) subjects [2]. In the present study, to evaluate the influence of height on progressive vascular remodeling, further analyses showed no-significant association between height and CIMT for subjects with both high and low hemoglobin; fully-adjusted standardized parameter estimates (β) and *p*-values were β = −0.001, *p* = 0.992 for subjects with high hemoglobin and β = 0.11, *p* = 0.290 for subjects with low hemoglobin. Since our present study population comprised individuals within the normal BMI range (18.5–24.9 kg/m^2^) our present results are compatible with those of previous studies we conducted [2, 5]. However, atherosclerosis (increased CIMT) is only one aspect of vascular repair (aggressive endothelial repair). Therefore, compared to subjects with a tall stature, subjects with a short stature may show much stronger vascular repair activity (appropriate endothelial repair) even within the normal BMI range.

Endothelial dysfunction has been recognized as one of the initial mechanisms leading to atherosclerosis (increased arterial stiffness) [18]. Platelets are the first circulating blood cells that interact with an injured vessel (injured endothelium) [19]. Platelets usually do not interact with the intact vascular endothelium. Once the arterial endothelium becomes injured, sub-endothelial components such as collagen [20] and von Willebrand factor [21] are exposed, resulting in the adherence of platelets to the damaged vessel wall and their subsequent activation. Activated platelets (P-selectin-positive platelets) release stromal cell-derived factor-1 (SDF-1) [22]. And co-cultivation experiments showed that human platelets recruit CD34-positive cells via specific adhesion receptors P-selectin/PSGL-1 and beta1- and beta2-integrins. This study also reported that platelets induced differentiation of CD34-positive cells into mature endothelial cells and foam cells [11]. Another study reported that Platelet-derived SDF-1 regulates adhesion of stem cells *in vitro* and *in vivo*, and the differentiation of CD34-positive cells to endothelial progenitor cells [10].

Since bone marrow-derived endothelial cells such as CD34-positive stem cells support the integrity of the vascular endothelium [23], platelets play an important role in vascular repair not only by serving as “bridging cells” between endothelial progenitor cells and the injured arterial endothelium, but also by inducing the differentiation of CD34-positive cells.

Furthermore, platelet-rich plasma could enhance the proliferation of bone marrow mesenchymal stem cells, which are known to be multi-potent stem cells [24]; and we also previously reported that the number of platelets indicates the activity of vascular repair [12]. Platelets not only indicate vascular repair activity but the degree of endothelial injury as well. In the present study, the positive association between platelets and circulating CD34-positive cells and CIMT of subjects with high hemoglobin might support the above-mentioned mechanism.

However, no such associations were found in subjects with a low hemoglobin level. Bone marrow activity decreases as individuals age [25], which may be associated with the increased frequency of anemia seen in the elderly [14], and bone metabolism is known to be associated with vascular maintenance [13]. In addition, hypertension is a strong endothelial impairment factor and hemoglobin has been revealed to be positively associated with hypertension [15], atherosclerosis [16], and hypertension-induced vascular damage [17]. Therefore, a weak vascular repair response is caused both by an age-related reduction in bone marrow activity and a small degree of vascular injury resulting in a low level of hemoglobin, which might act as a strong confounding factor on platelets as an indicator of vascular remodeling.

**Figure 2:**
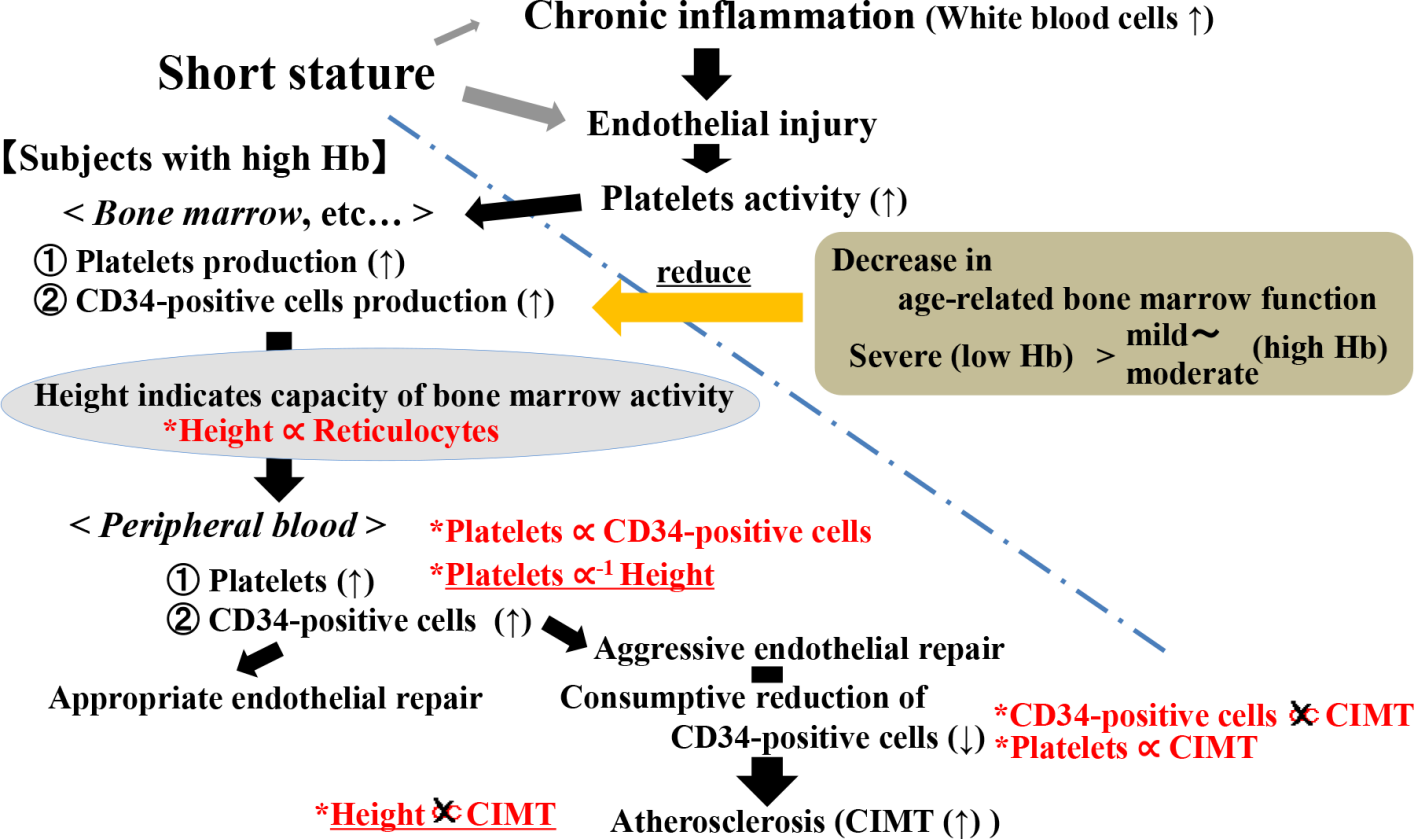
Possible mechanism underlying the association between height and vascular remodeling (endothelial repair) The mechanism below the dotted line is observed only for subjects without severe age-related bone marrow activity reduction (subjects with high Hb). Relations in red were observed in the present analysis. Hb: hemoglobin. CIMT: carotid intima-media thickness.

A summary of the possible mechanisms underlying the association between height and vascular remodeling (endothelial repair) is shown in Figure 2. Short stature entails a higher risk of endothelial impairment [2] such as chronic inflammation [5, 6], which stimulates endothelial repair activity. Since endothelial activity is closely associated with bone marrow activity [13, 23], which is also strongly influenced by age-related decline [14, 25], an analysis limited to subjects with high bone marrow activity (high hemoglobin) should strengthen the influence of endothelial repair. However, although both platelet and CD34-positive cell production are stimulated by endothelial injury, aggressive endothelial repair (the cause of atherosclerosis) may cause consumptive reduction of CD34-positive cells, but not platelets [26]. Therefore, in the present study, among subjects with high hemoglobin, although a significant positive association between platelets and CIMT was observed, there was no significant association between circulating CD34-positive cells and CIMT. In addition, as in our previous study [2], we found no significant association between height and CIMT. Additional studies we conducted previously reported that a high level of platelets and a low level of circulating CD34-positive cells is an independent risk factor for the vicious cycle between hypertension and atherosclerosis, whereas high platelets and high CD34-positive cells are not since appropriate endothelial repair is established in these subjects [26]. In our present study, we found a significant positive association between platelets and circulating CD34-positive cells among subjects with high hemoglobin levels. The majority of these subjects with high hemoglobin may therefore activate endothelial repair activity and establish appropriate endothelial repair, resulting in no significant association between height and CIMT. Further investigations with a larger number of subjects with high hemoglobin and circulating CD34-positive cell data that enables us to perform circulating CD34-positive cell level-specific analysis will be necessary. In addition to these mechanisms, in our present study, we found a significant positive association between height and reticulocytes among subjects with a high hemoglobin concentration, as shown in a previous study [27]. Since endothelial activity is strongly associated with bone marrow activity [13, 23] and reticulocytes act as an indicator of such activity, this positive association between height and reticulocytes may also partly indicate that subjects with a short stature might be at a disadvantage as a result of deficient endothelial repair.

In the present study, although the correlation between height and platelet count is small, short stature activates vascular remodeling more strongly than tall stature for following reasons. First, height is positively associated with vascular maintenance capacity [28]. If aggressive vascular repair is evoked by serious endothelial damage, the maximum ability of vascular repair should be defined by height—subjects with a short stature would not activate vascular repair as strongly as subjects with a tall stature (ceiling effect). Since short stature is associated with inflammatory disadvantages [5, 6], this type of ceiling effect should act as a strong confounding factor on the association between height and platelet count. We also previously reported that height is positively associated with hematopoietic capacity [27], and bone marrow stroma cells differentiate into megakaryocytes that subsequently produce platelets [29]. In the present study population, reticulocytes were found to be significantly positively associated with height. Therefore, hematopoietic capacity also should act as a strong confounding factor on the association between height and platelet count.

In previous study, a significant positive correlation was reported between height and number of circulating CD34-positve cells in subjects with systolic hypertension but not in those without, independent of known cardiovascular risk factors [28]. And we also reported in previous study that the number of platelets and circulating CD34-positive cells acts as an indicator of the activity of the vicious cycle that exists between hypertension and endothelial dysfunction in elderly Japanese men [26]. Since a significant inverse association was observed between height and platelet count among elderly Japanese men in present study, those with short stature might also activates the vicious cycle that exists between hypertension and endothelial dysfunction much more strongly compared to those with a tall stature. Since hypertension, but not height, can be altered by improving living habits, it is necessary that subjects with a short stature control their daily blood pressure much more precisely compared to those with a tall stature.

Although our present study employs a small sample size, it is the largest study in the world that deals with circulating CD34-positive cells among the general elderly population who are selected in a strict manner—subjects were restricted to men in a narrow age and normal BMI range because gender differences, age and high BMI can act as strong confounding factors on the association between height and other variables [30–32].

Potential limitations of this study warrant consideration. Because creatinine clearance data were not available and estimated glomerular filtration rate (GFR) is not an effective tool for evaluating kidney function for a comparison of associations with various body heights [1, 2, 5, 27, 28, 32], we were not able to perform an analysis adjusted for exact renal function. However, our study showed that the association between height and platelet count remained significant even after adjustment for serum creatinine. Although significant associations exist between height and hematological parameters such as platelets and reticulocytes, no data was available with regard to the evaluation of endothelial function. Further analyses that include endothelial function-related data such as Flow Mediated Dilation (FMD) will be necessary. Additionally, because this was a cross-sectional study, causal relationships were not able to be established. However, since height was regarded as a surrogate marker of childhood social and physical condition [1,2,5,6,27,28,32] and the target population in the present study was elderly men, we believe this investigation has the characteristics of a prospective study to a certain degree. Further prospective population based studies are needed to eliminate the possibility of a causal relationships.

In conclusion, a significant inverse association was observed between height and platelet count, while a significant positive association was seen between platelets and circulating CD34-positive cells and CIMT in subjects with a high hemoglobin concentration, but not in those with a low hemoglobin concentration. These results indicate that among elderly Japanese men, those with a short stature activate vascular remodeling much more strongly compared to those with a tall stature.

## MATERIALS AND METHODS

### Ethics statement

Participants in this study was essentially voluntary. Written consent forms were available in Japanese to ensure comprehensive understanding, and informed consent was given by all participants. This study was approved by the Ethics Committee for Human Use of Nagasaki University (projects registration number 14051404).

### Study populations

To avoid the influence of age on height, this study was comprised of subjects in a narrow age range, as in a previous study that reported the influence of height among elderly subjects [27, 28]. The original population included 409 men 65 to 69 years old residing in rural communities in Nagasaki Prefecture in western Japan. Participants were recruited from 2013–2015. To avoid the influence of inflammatory and hematological disease, subjects with high and low white blood cell counts (≥ 10,000 cells/μL (*n* = 2) and 1,000 cells/μL< (*n* = 1), respectively) were excluded. Additionally, to avoid the influence of medication activating the bone marrow, subjects taking medication for anemia (*n* = 3) were also excluded.

Since hemoglobin value shows a strong positive correlation with body mass index [15, 16], and another study reported a J- or U-shaped correlation between BMI and mortality [33], abnormal BMI status might act as a strong confounding factor for the correlation between height and reticulocyte count. Therefore, to avoid the influence of undernutrition and hyper-nutrition, subjects with a BMI < 18.5 kg/m^2^ (*n* = 50) and BMI ≥ 25 kg/m^2^ (*n* = 103), respectively, were excluded. Subjects with no evaluable laboratory data (*n* = 1) and with no CD34-positive cell count data available (*n* = 30) were also excluded, leaving a total of 219 subjects participating in the study.

### Data collection and laboratory measurements

Trained interviewers obtained information on medical history. Body weight and height of patients wearing light clothing were measured using an automatic body composition analyzer (BF-220; Tanita, Tokyo, Japan), and BMI (kg/m^2^) was calculated. Fasting was defined as not taking breakfast. Fasting blood samples were collected in a heparin sodium tube, EDTA-2K tube and a siliconized tube. Fresh samples (within 24 hours from drawing) from the heparin sodium tube were used to determine the number of CD34-positive cells. BD (Beckton Dickinson Biosciences) Trucount^TM^ technology, an accurate and reproducible single platform assay cited in the International Society of Hematotherapy and Graft Engineering (ISHAGE) guidelines [34] and supported by automated software on the BD FACSCant^TM^ II system, was used to measure circulating CD34-positive cells.

Samples from the EDTA-2K tube were used to measure white blood cell count, platelet count and reticulocytes using automated procedure at SRL, Inc. (Tokyo, Japan). Serum triglycerides, serum high density lipoprotein (HDL) cholesterol, serum aspartate aminotransferase (AST), serum γ-glutamyltranspeptidase (γ-GTP), hemoglobin A1c (HbA_1C_), and serum creatinine were measured using standard laboratory procedures at SRL, Inc. (Tokyo, Japan).

Measurement of CIMT by ultrasonography of the left and right carotid arteries was performed by an experienced vascular technician using a LOGIQ Book XP with a 10-MHz transducer, GE Healthcare, Milwaukee, WI, USA). Mean values for the left and right CIMT were calculated using automated digital edge-detection software (Intimascope; MediaCross, Tokyo, Japan), with the protocol described in detail elsewhere [35].

### Statistical analysis

Characteristics of the study population stratified by hemoglobin levels were expressed as mean ±standard deviation. Simple and partial correlation analysis (Pearson) adjusted for known cardiovascular risk factors were performed to evaluate hematological parameters, CIMT and height. Simple correlation coefficients of platelets and other variables were calculated. Multiple linear regression analyses were also performed to evaluate the same. Since inter-correlation with systolic blood pressure was *r* = 0.72 (*P* < 0.001), diastolic blood pressure was not analyzed as a confounding factor. Because circulating CD34-positive cells, triglycerides, γ-GTP, and serum creatinine had a skewed distribution, logarithmic transformation was performed for the simple and partial correlation analysis, and linear regression analysis. All statistical analyses were performed with the SAS system for Windows (version 9.4; SAS Inc., Cary, NC). Probability values of less than 0.05 were considered to be statistically significant.
